# The prevalence and characteristics of chronic ocular itch: a cross-sectional survey

**DOI:** 10.1097/itx.0000000000000004

**Published:** 2017-03-28

**Authors:** Carolyn Stull, Rodrigo Valdes-Rodriguez, Brian M. Shafer, Alina Shevchenko, Leigh A. Nattkemper, Yiong-Huak Chan, Sydney Tabaac, Martin J. Schardt, Dany M. Najjar, William J. Foster, Gil Yosipovitch

**Affiliations:** Departments of a Dermatology; b Ophthalmology, Lewis Katz School of Medicine at Temple University, Philadelphia, PA; c Biostatistics Unit, Yong Loo Lin School of Medicine, National University of Singapore, Singapore, Singapore; d Department of Dermatology and Itch Center, University of Miami Miller School of Medicine, Miami, FL

**Keywords:** Pruritus, Allergic conjunctivitis, Keratoconjunctivitis sicca, Dry eye syndrome, Blepharitis, Atopic dermatitis, Antihistamine

## Abstract

**Introduction:**

In this cross-sectional study, we aimed to determine the prevalence and characteristics of chronic ocular itch in an outpatient ophthalmology and optometry clinic.

**Methods:**

Four hundred patients from an outpatient ophthalmology and optometry clinic were enrolled. The presence and characteristics of chronic ocular itch were assessed by a questionnaire. Data regarding ophthalmologic, dermatologic, and systemic conditions as well as current medications were extracted from medical records.

**Results:**

Chronic ocular itch was present in 118 (29.5%) of 400 participants. Chronic ocular pruritus was significantly more prevalent in females [*P*=0.015; odds ratio (OR)=1.8; 95% confidence interval (CI), 1.1–2.8] and was significantly associated with the presence of allergic conjunctivitis [51.8% (n=45); *P*<0.001; OR=5.0; 95% CI, 3.0–8.3], dry eye syndrome [40.1% (75); *P*<0.001; OR=2.6; 95% CI, 1.7–4.1], blepharitis [43.8% (n=21); *P*=0.021; OR=2.0; 95% CI, 1.1–3.8], and atopic dermatitis [50.0% (n=10); *P*=0.023; OR=2.6; 95% CI, 1.1–5.8]. Chronic ocular itch was not significantly associated with systemic conditions, or the use of prescribed ophthalmologic medications.

**Discussion:**

Chronic ocular itch is common and may be related to ophthalmologic or dermatologic pathologies. The present findings highlight the importance of identifying and managing this uncomfortable symptom that may negatively impact the quality of life and sleep of affected patients.

## Introduction

Ocular itch (pruritus) is a common, uncomfortable, and bothersome sensation. This symptom is frequently reported in the context of ocular pathology or eyelid dermatitis and has numerous etiologies. Ocular itch is considered a hallmark feature of allergic conjunctivitis (AC), but may also occur in other ophthalmologic conditions such as dry eye syndrome (DES) and blepharitis^1^. In addition, ocular itch may arise from dermatologic conditions that affect the eyelids, including atopic dermatitis (AD), allergic contact dermatitis, and irritant contact dermatitis^2^,^3^. Furthermore, ocular itch may occur as an adverse reaction to ophthalmologic medications including prostaglandin analogs and antibiotics, or preservatives^4^–^6^.

Despite its prevalence, the pathogenesis of ocular itch remains poorly understood. Previous studies have focused largely on the acute, allergic variant of ocular itch. However, ocular itch may also be nonallergic in origin, and may persist for months to years as a chronic condition.

The aim of this study was to determine the prevalence and characteristics of chronic ocular itch, and to elucidate associations between chronic ocular itch and ophthalmologic, dermatologic, and systemic conditions. These findings will improve our understanding of this entity, and increase recognition of this common and disruptive symptom.

## Methods

### Participants, sample size, and study setting

In this cross-sectional study, we enrolled subjects from Temple University’s ophthalmology and optometry outpatient clinic. All English-speaking individuals over the age of 18 were eligible for participation. Individuals were approached without any prior knowledge of ophthalmologic conditions or comorbidities. Of 460 individuals who were approached, 400 elected to participate. The protocol for this study was approved by the local Institutional Review Board, and all study participants provided voluntary signed informed consent. Subjects were enrolled from December 2014 through October 2015.

### Questionnaire

The presence and characteristics of chronic ocular itch were assessed with a questionnaire. The questionnaire was modified from one used in previous studies of chronic itch with proven reliability, validity, and internal consistency^7^. The questionnaire contained 2 sections. The first recorded demographic information, and was completed by all 400 subjects. The second was completed only by patients with chronic ocular itch, and assessed the history and characteristics of itch. In accordance with the classification of chronic itch outlined by the International Forum for the Study of Itch, we defined chronic ocular pruritus as itch lasting ≥6 weeks^8^. Participants recorded the duration of pruritus, and the frequency of pruritic episodes. Subjects specified symptoms that accompanied itch, factors that exacerbated itch, and factors that relieved itch. Subjects described the characteristics of their itch by selecting applicable descriptions from a list of 45 options. In addition, participants specified whether they experience itch during the day or at night. Participants rated their itch intensity on a numeric rating scale (NRS) with end points labeled 0=“no itch” and 10=“unbearable itch.” The NRS has been shown to be valid and reliable in individuals with chronic itch^9^. Participants rated the pleasurability of scratching or rubbing their eyes in response to itch on a scale with end points labeled −5=“highly unpleasurable” and 5=“highly pleasurable.” Participants indicated the location of their pruritus by circling the affected area on a depiction of the ocular region.

### Review of medical records

Data regarding ophthalmologic, dermatologic, and systemic diagnoses, as well as current medications, were extracted from patient medical records.

### Data analysis

All statistical analyses were performed using PASW 18.0 software (SAS, Chicago, IL) with statistical significance set at *P*<0.05. Descriptive statistics for quantitative variables were presented as mean±SD and as percentages for qualitative variables. Differences in quantitative outcomes were assessed using parametric tests when normality and homogeneity assumptions were satisfied; otherwise the equivalent nonparametric tests were used. Associations between categorical variables were determined using χ^2^ or Fisher Exact tests; with odds ratios (ORs) presented where applicable. Correlations were assessed using the nonparametric measure of the Spearman rank correlation.

## Results

### Demographic factors

The mean age of the 400 subjects was 58 years (SD, 16.1; range, 18–93 y). Females composed 252 (63%) of participants, and males composed the remaining 148 (37%). Subjects were identified as African American 242 (60.5%), white 91 (22.8%), Hispanic/Latino 44 (11.0%), Asian 15 (3.8%), and other 8 (2.0%). Demographic features of subjects with chronic ocular itch are described in **Table** **1**.

**Table 1:**
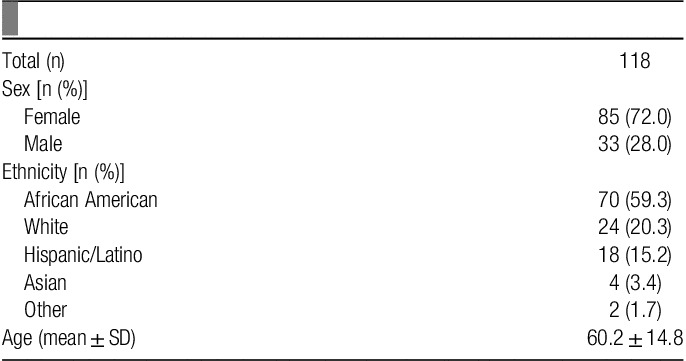
Demographic features of subjects with chronic ocular itch.

### Prevalence, characteristics, and associations

Chronic ocular itch was reported by 118 (29.5%) of 400 individuals and was significantly associated with female sex [*P*=0.015; OR=1.8; 95% confidence interval (CI), 1.1–2.8]. The mean NRS itch intensity was 5.6±2.6. NRS intensity was significantly correlated with duration of itch (*r*=0.19, *P*<0.05). The overall mean duration of chronic itch was 2 years (range, 6 wk to 58 y). The mean length of a single episode of itch was 4 hours (range, 2 s to 7 d). Chronic itch was experienced more frequently during the day [87.3% (n=103)] than at night [63.6% (n=75)]. The mean pleasurability of scratching/rubbing the pruritic area was 1.9±2.9, and was positively correlated with itch intensity (*r*=0.18, *P*<0.05). Itch occurred concurrently with tearing [54.2% (n=64)], and pain [24.6% (n=29)]. Itch was described as burning [53.4% (n=63)], throbbing [28.0% (n=33)], sharp [31.4% (n=37)], and tingling [49.2% (n=58)]. Exacerbating factors included weather and seasonal changes [43.2% (n=51)], and exposure to allergens [41.5% (n=49)]. Pruritus was described as unbearable [33.1% (n=39)], torturing [27.1% (n=32)], life-restricting [26.3% (n=31)], and disruptive to sleep [17.8% (n=21)]. Factors that relieved pruritus included the use of artificial tears [44.9% (n=53)], cool compress [25.4% (n=30)], warm compress [16.9% (n=20)], and antihistamine eye drops [12.7% (n=15)]. Commonly indicated locations of chronic ocular pruritus included the inner canthi [20.3% (n=24)], lower eyelids [20.3% (n=24)], and upper eyelids [19.5% (n=23)].

Ophthalmologic conditions significantly associated with chronic ocular pruritus included AC [51.8% (n=45); *P*<0.001; OR=5.0; 95% CI, 3.0–8.3], DES [40.1% (n=75); *P*<0.001; OR=2.6; 95% CI, 1.7–4.1], and blepharitis [43.8% (n=21); *P*=0.021; OR=2.0; 95% CI, 1.1–3.8] (**Table** **2**). Dermatologic conditions significantly associated with chronic ocular pruritus included AD [50.0% (n=10); *P*=0.023; OR=2.6; 95% CI, 1.1–5.8] and the presence of chronic cutaneous pruritus (*P*<0.001; OR=3.7; 95% CI, 2.3–6.0). Chronic ocular itch was not significantly associated with the presence of anxiety, depression, or any other systemic disease. There were no statistically significant relationships between chronic ocular itch and the use of prescribed ophthalmologic medications.

**Table 2:**
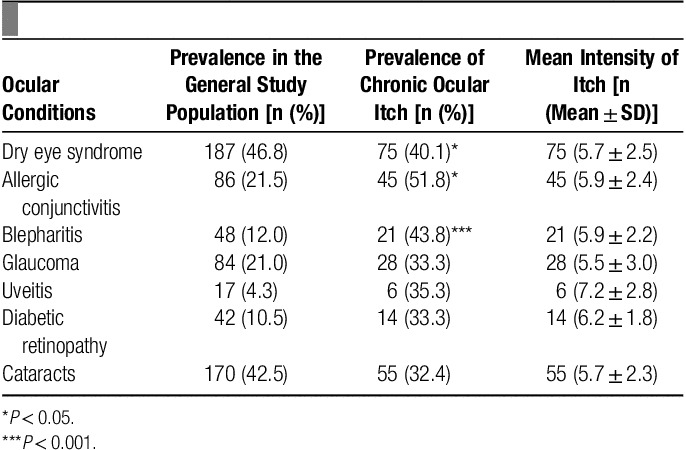
Prevalence of ophthalmologic conditions, chronic ocular itch, and itch intensity.

## Discussion

The present findings demonstrate that chronic ocular pruritus is a common symptom affecting 29.5% of patients in our study population. Notably, this number far exceeds the reported prevalence of chronic cutaneous pruritus, 13.5%^10^. In addition, our findings revealed the average NRS itch intensity to be moderately high (5.6). Itch intensity correlated with duration of itch, suggesting that pruritus may become more severe over time. It is plausible that progressive neural hypersensitization, a phenomenon implicated in many etiologies of chronic itch, may underlie such findings^11^. In addition, itch intensity positively correlated with the degree of pleasure derived from scratching or rubbing the affected area. This finding has also been reported in other types of chronic itch including AD^12^.

Interestingly, subjects experienced itch more frequently during the day than at night. This finding runs contrary to what has been previously reported in other etiologies of chronic itch. The factors underlying this outcome remain unclear. However, it is possible that the closure of the eyelids may provide a protective barrier, reducing tear film evaporation and dampening sensory pruritogenic stimuli from the environment.

In addition, our results indicate that chronic ocular pruritus may have a life-restricting and sleep-disrupting impact. Our findings suggest that the burden of chronic ocular pruritus is high, based on the number of patients who used terms including “unbearable” and “torturing” to describe their itch. Given the severity of these findings, further evaluation of the impact of chronic ocular itch on quality of life would be of great interest.

Chronic ocular pruritus was associated with both allergic and nonallergic ophthalmologic pathologies. The condition most strongly associated with chronic ocular itch was AC, which is estimated to affect up to 40% of the general population^13^. Although itch has long been described as the cardinal symptom of this disease, we found that pruritus commonly persists as a chronic complaint. Ocular itch associated with AC can be severe and significantly interfere with daily activities^14^. It is likely that chronic itch is also prevalent in chronic variants of AC, including atopic keratoconjunctivitis, and vernal keratoconjunctivitis. However, due to the low prevalence of these conditions in our study population, we were unable to assess these associations.

Our results revealed that chronic ocular pruritus is also common in the setting of DES, also known as keratoconjunctivitis sicca, a highly prevalent condition within our study population. A previous study demonstrated a high association between patient-reported symptoms of itch and dryness^1^. However, the significance of pruritus in the setting of DES has rarely been studied. Indeed, validated questionnaires used to evaluate the severity of DES do not universally assess for the presence and severity of ocular itch. On the basis of our findings, which indicate that pruritus is both prevalent and burdensome, we suggest that ocular itch be assessed in these patients. In addition, it is worth noting that DES has many etiologies including, but not limited to, blepharitis and lacrimal gland dysfunction. Furthermore, DES may occur in the context of systemic diseases such as Sjogren’s syndrome, rheumatoid arthritis, and systemic lupus erythematosus. Future studies could elucidate the variation in prevalence and characteristics of ocular itch among different etiologies of DES.

Our findings additionally revealed a high association between chronic ocular pruritus and blepharitis, or meibomian gland dysfunction. Blepharitis is clinically characterized by swollen, inflamed lid margins, and has been described as a complication of various pruritic dermatoses, including AD, seborrheic dermatitis, and psoriasis^2^,^15^. Itch has been previously described as a symptom of blepharitis; however, little is known about its pathogenesis^16^.

A significant association was also revealed between chronic ocular pruritus and AD. Pruritus is pathognomonic of AD, in which a vicious itch-scratch cycle often perpetuates the disease. Eyelid involvement is common in patients with AD, affecting >10% of individuals^2^. In addition, AD also imparts increased risk of pruritic ophthalmologic pathologies, including AC and blepharitis.

Despite its prevalence, the pathophysiology underlying various forms of chronic ocular pruritus remains poorly understood. Consequently, it comes as no surprise that currently available treatment regimens have limited efficacy. Pharmacologic intervention has largely targeted histamine with use of H_1_ antagonists and mast cell stabilizing agents, especially in the context of ocular allergy. While histamine has known involvement in AC itch, an additional histamine-independent mode of itch induction has recently been elucidated^17^. In a murine model, Huang et al^18^ demonstrated that both TRPV1, a histamine-sensitive ion channel, and TRPA1, a histamine-independent ion channel are required for the induction of allergic ocular itch. This finding may explain the limitations of currently available anti-allergy eye drops^19^.

The present study has a number of limitations. First, the cross-sectional design of our study precludes the establishment of causal relationships. Another limitation is the possibility that various ophthalmologic conditions, including blepharitis, may be underrepresented in our study population. The nationwide prevalence of blepharitis has been reported to range from 37% to 47%, which exceeds the 12% prevalence in our study population^20^. This discrepancy could be explained by a number of contributing factors. It is possible that in some incidental cases the presence of blepharitis may not have been recorded. However, this finding could also be reflective of our predominantly African American study population, as blepharitis may be less prevalent in such individuals. Lastly, the size of our study population precluded meaningful assessment of chronic ocular itch in ophthalmologic conditions with low prevalence. There are a number of aspects of chronic ocular itch that would be interesting to explore in future studies. These include the distinguishing characteristics of itch within different conditions, as well as a thorough investigation of treatment response.

In conclusion, chronic ocular itch is a prevalent symptom that is associated with AC, DES, blepharitis, and AD. In clinical practice, increased recognition of this distressing symptom, and further exploration of its causes, would be of great benefit to patients.
